# An improved peptide-spectral matching algorithm through distributed search over multiple cores and multiple CPUs

**DOI:** 10.1186/1477-5956-12-18

**Published:** 2014-04-11

**Authors:** Jian Sun, Bolin Chen, Fang-Xiang Wu

**Affiliations:** 1 Division of Biomedical Engineering, University of Saskatchewan, 57 Campus Dr, S7N 5A9 Saskatoon, SK, Canada; 2 Department of Mechanical Engineering, University of Saskatchewan, 57 Campus Dr, S7N 5A9 Saskatoon, SK, Canada

## Abstract

**Background:**

A real-time peptide-spectrum matching (RT-PSM) algorithm is a database search method to interpret tandem mass spectra (MS/MS) with strict time constraints. Restricted by the hardware and architecture of individual workstation, previous RT-PSM algorithms either are not fast enough to satisfy all real-time system requirements or need to sacrifice the level of inference accuracy to provide the required processing speed.

**Results:**

We develop two parallelized algorithms for MS/MS data analysis: a multi-core RT-PSM (MC RT-PSM) algorithm which works on individual workstations and a distributed computing RT-PSM (DC RT-PSM) algorithm which works on a computer cluster. Two data sets are employed to evaulate the performance of our proposed algorithms. The simulation results show that our proposed algorithms can reach approximately 216.9-fold speedup on a sub-task process (similarity scoring module) and 84.78-fold speedup on the overall process compared with a single-thread process of the RT-PSM algorithm when 240 logical cores are employed.

**Conclusions:**

The improved RT-PSM algorithms can achieve the processing speed requirement without sacrificing the level of inference accuracy. With some configuration adjustments, the proposed algorithm can support many peptide identification programs, such as X!Tandem, CUDA version RT-PSM, etc.

## Background

Tandem mass spectrometry (MS/MS) has been widely used in the early detection of diseases, chemical analysis and pharmaceutical industry. It can efficiently identify and characterize the protein component information in complex biological mixtures. Interpretations of MS/MS spectra need to perform peptide-spectrum matches (PSMs) by searching experimental MS/MS spectra against a protein sequence database.

In order to improve the efficiency and the accuracy of MS/MS experiments, a real-time peptide identification procedure needs to be involved in a mass spectrometry system which analyzes peptides and performs the PSMs in a peptide identification procedure life-circle. Wu et al. [1] have proposed a pretty fast procedure, called real-time PSM (RT-PSM). The key component is “identifying peptides”, which is performed by a software application [1]. However, this RT-PSM procedure does not include any external software controlling features. Although the method is fast, further experiments indicate that the programming still cannot completely satisfy all real-time system requirements, since it is a single-thread program that runs on a single workstation.

As a real-time system, the time window of each peptide identification procedure is limited by the spectrum acquiring time of mass spectrometers. It could be between 0.05 second to 0.5 second due to different mass spectrometers. To fit in the narrow time window, using parallel computation to improve the speed of PSMs is becoming a trend. Duncan et al. [2] develop a program called Parallel Tandem by using a computer cluster. It processes MS/MS in parallel by using X!Tandem and a computer cluster with Parallel Virtual Machine (PVM) or Message Passing Interface (MPI). Sadygov et al. [3] develop the parallel version of SEQUEST, which is also based on the PVM in a computer cluster. Diament et al. [4] further develop a faster SEQUEST, called Tide, to speed up the performance of the SEQUEST. It acheives up to 170 times faster than SEQUEST. Zhang et al. [5,6] use SIMD instructions in a single workstation to develop programs for improving the speed of peptide identification procedures. Graumann et al. [7] recently develop a framework of intelligent agent, termed MaxQuant Real-Time, which is implemented in the MaxQuant computational proteomics environment. The framework is especially uesful for new instrument types, such as the quadrupole-Qrbitrap.

No matter using a computer cluster or a single workstation, the principles of parallel computing are identical: dividing a large sequential process into several independent sub-processes and executing the sub-processes concurrently to reduce the execution time [8]. However, those previous parallel computing methods [1-7] still have some room to be improved. In terms of processing time, parallel forms of X!Tandem [2] and SEQUEST [3] spend more time than the RT-PSM algorithm proposed in [1] when analyzing individual spectra. Although the Tide [4] is already very fast, the speed can still be improved. In terms of computing environments, SIMD instructions are restricted by the CPU L2 Cache [9,10], which often needs to sacrifice the level of inference accuracy to achieve the time limitation of a real-time system, while a computer cluster circumvents this problem. Moreover, instead of design a specific program, we aim to develpe a general platform that can support many peptide identification programs.

In this paper, we develop an improved peptide identification procedure on a computer cluster based on the RT-PSM algorithm proposed by Wu et al. in [1]. Two parallel algorithms are developed in this study: a multi-core RT-PSM algorithm (MC RT-PSM) which works on an individual workstation in form of a multi-thread program and a distributed computing RT-PSM algorithm (DC RT-PSM) which works on a computer cluster in form of a distributed computing program. The DC RT-PSM is built by using the parallelized MC RT-PSM procedure, which allocates and manages task computating resources through a head node in the distrubuted computing procedure. Source code of the DC RT-PSM algorithm and sample data are available in the Additional file 1. The improved algorithms can achieve processing speed requirements without sacrificing the level of inference accuracy.

## Results and discussion

### Experimental environment and data sources

The experimental computer cluster consists of one head node and 32 worker nodes, which is connected with 1 Gigabit Ethernet. Each node has 8 logical CPU cores.

Two datasets are employed to test the improved algorithms in this study. Dataset A is the one used in the RT-PSM package [1]: the MS/MS spectrum experimental data source includes 2058 group spectrum data and the protein database is taken from a subset of the UniRef100 human protein database. It contains over 2200 entries (over 180000 peptide sequences). Dataset B includes 16463 groups of experimental spectrum data and over 3300 entries. It is also generated from the UniRef100 human protein database.

### The level of inference accuracy for the improved algorithms

The purpose of the parallel computing processing in our improved algorithms is to reduce the peptide identification time. Hence, the proposed algorithms do not gain better performance by decreasing the level of inference accuracy. The results of the improved algorithms should be identical with the original RT-PSM in [1]. We randomly choose 100 groups of experimental data from the results of our improved alogirthms and the original RT-PSM. The identification results are in excellent agreement between the original RT-PSM program and our improved MC RT-PSM and DC RT-PSM programs.

### The time speedup of the 2-Dimensional peptide database search method

Rather than using binary search method to query the peptide sequence database, we propose to employ the 2-Dimensional peptide database search method. Although this new search method does not improve the accuracy of candidate peptide selection, it can speed-up the procedure to a certain degree. For example, in Dataset A, the new search method makes the similarity-scoring module spent less than 6.7% execution time. The detail information of time spending is shown in Table 1.

**Table 1:** **Comparisons between the binary search method and the 2**-**dimensional peptide database search method**

**Method**	**Spectra number**	**Time (ms)**
Binary search method	2058	8.043
2-Dimensional peptide database search method	2058	7.668

### The time speedup of the MC RT-PSM procedure

As expected, the performance of MC RT-PSM mainly depends on the speed of CPU frequency and the number of logical cores of the CPU. The MC RT-PSM is tested on four different computers. Table 2 shows the detail information of those computers’ CPUs.

**Table 2:** The detail information of experiment hardware environments

**Name**	**CPU**	**Threads**	**HT**	**Usage**
WS1	i7 3770	8	YES	Personal server
WS2	i5 750	4	NO	Development PC
WS3	XEON E5410	8	NO	Worker node of cluster
WS4	i7 2720QM	8	YES	Personal computer

**Figure 1:**
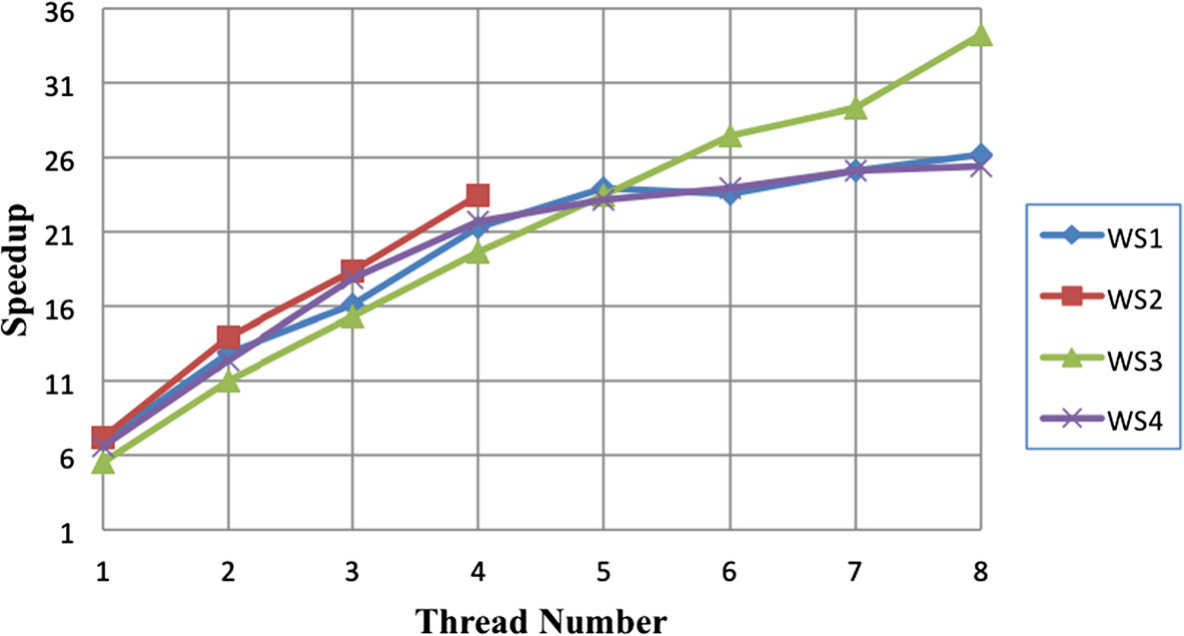
**The speedup of execution time of the similarity-scoring module for the MC RT-PSM compared with RT-PSM algorithm proposed by Wu et al. **[1] **in four experiment computers.**

In terms of the time speedup, we compare the MC RT-PSM with the RT-PSM proposed by Wu et al. in [1]. Figure 1 illustrates the time speedup of the MC RT-PSM procedure. The numerical experiments are conducted on the same dataset (Dataset A). When using one single-thread, the MC RT-PSM achieves about 5-fold speedup than the RT-PSM proposed by Wu et al. [1]. When increasing the thread number to eight, the MC RT-PSM achieves about 25 to 34-fold speedup than the RT-PSM procedure.

### The time speedup of the DC RT-PSM procedure

The performance of the DC RT-PSM is compared with the single-thread MC RT-PSM procedure. Two tasks are designed for comparisons. Task 1 is to search 2058 spectra against 2200 protein entries that conducted on Dataset A. Task 2 is to search 16463 spectra against 3300 protein entries that conducted on Dataset B.

**Figure 2:**
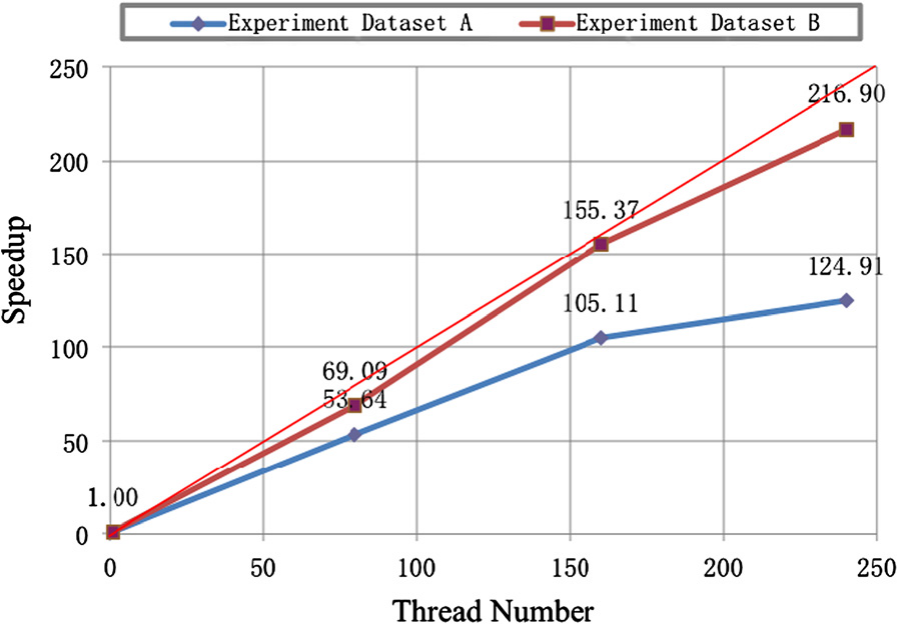
**The speedup of execution time of the similarity-scoring module for the DC RT-PSM compared with MC RT-PSM algorithm in two experiment databases.**

The comparison is first done on the similarity-scoring module, which is the core part of those algorithms. For taks 1, the DC RT-PSM is 53.64-fold speedup with 80 threads (10 worker nodes), 105.11-fold speedup with 160 threads (20 worker nodes) and 124.91-fold speedup with 240 threads (30 worker nodes) compared with the single-thread MC RT-PSM program. For taks 2, the DC RT-PSM is 69.09-fold speedup with 80 threads, 155.37-fold speedup with 160 threads and 216.90-fold speedup with 240 threads compared with the single-thread MC RT-PSM program. The results are shown in Figure 2. Generally, an ideal parallel computing algorithm is able to gain k-fold speedup when a task is allocated into k threads. The performance of DC RT-PSM is close to the theoretical performance, especially when it is used to search a large scale database (such as task 2 in our experiments), which is quite promising.

**Figure 3:**
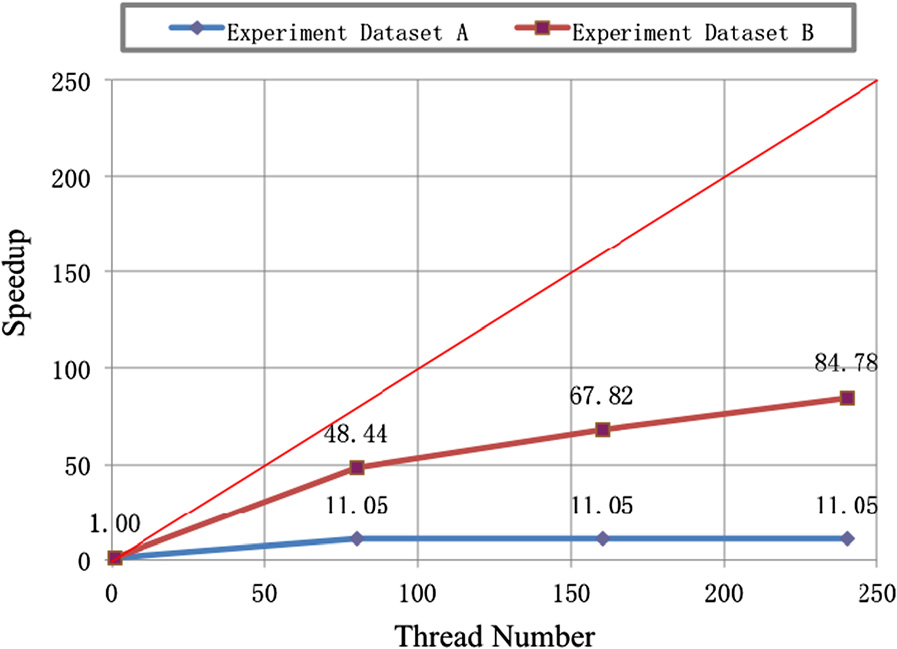
**The overall speedup of execution time for the DC RT-PSM compared with MC RT-PSM algorithm in two experiment databases.**

The comparison is then done on the overall performance of those programs. For task 1, no matter whether 80, 160 or 240 threads are allocated, the whole time spent by the DC RT-PSM is about 11-fold speedup compared with the single MC RT-PSM program. For task 2, the DC RT-PSM is 48.44-fold speedup with 80 threads, 67.82-fold speedup with 160 threads and 84.78-fold speedup with 240 threads compared with the single-thread MC RT-PSM program. The results are shown in Figure 3. The decreased fold speedup of the overall performances of DC RT-PSM is due to the fact of system natures. In the DC RT-PSM program, the task initial time and node message communication time are fixed, even worker nodes are connected with 1 Gigabit Ethernet. The time spent by those processes is about 2.0 seconds to 2.3 seconds. Therefore, if the experimental spectrum dataset is too small, the number of nodes allocated in a task could barely affect the total execution time, just like the performance of task 1 shows in Figure 3. Even though, the time speedup from DC RT-PSM algorithm is still promissing that can satisfies almost all real-time system requirements.

## Conclusions

In this paper, we have proposed an MC RT-PSM algorithm which works on an individual workstation and a DC RT-PSM algorithm which works on a computer cluster for interpreting MS/MS spectra. The MC RT-PSM algorithm is an extension of the single-thread RT-PSM algorithm proposed by Wu et al. in [1], while the DC RT-PSM algorithm is a distributed parallel computing algorithm that allocates and manages cluster worker nodes to perform the MS/MS spectrum analysis.

One advantage of our proposed method is that it is a general platform of parallel computing, since many current parallel algorithms are either not fast enough for all real-time MS/MS systems or restricted to specific computing environments. The distributed computing algorithm is designed not only for this RT-PSM algorithm but also for other similar algorithms. It can support many other peptide identification programs with some configuration adjustments, such as X!Tandem, SEQUEST, SIMD version RT-PSM, etc.. The other advantage of our method is that it can speed up the searching time. The proposed DC RT-PSM algorithm can reach the real-time constraints of most MS/MS systems without sacrificing the level of inference accuracy.

## Methods

### The performance of the RT-PSM program

**Figure 4:**
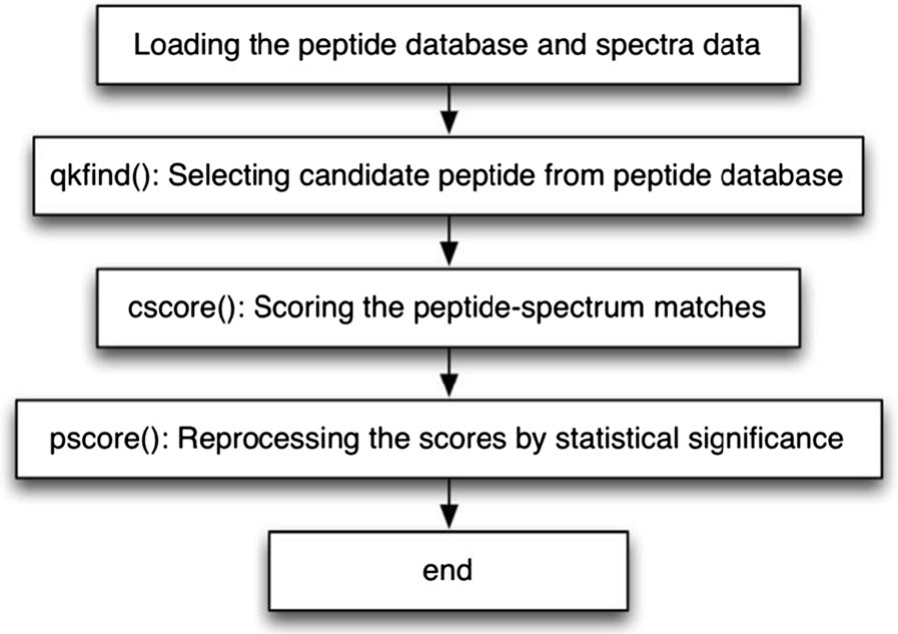
**The workflow of the RT**-**PSM algorithm proposed by Wu et al. **[1]**.**

The RT-PSM program proposed by Wu et al. [1] is a single-thread program. It contains four main steps. The first step is to load the peptide database and raw experimental spectrum data. The second step is to select candidate peptides from the peptide database. The masses of candidate peptides are those in the range of the experimental spectrum. Once a group of candidate peptides are selected, scores of every peptide-spectrum pairs are calculated in the third step (the similarity-scoring module). In the last step, after the program computes the statistical significance of the highest similarity score for each group, the final results are displayed. The workflow of the RT-PSM algorithm is shown in Figure 4.

The similarity-scoring module is the most time-consuming part in the RT-PSM program. It consumes over 95% CPU time in profiling experiments [5]. This is due to the fact that each spectrum has to be compared with the whole set of candidate peptides, which could easily contain thousands of peptide sequences. Hence, it is critical to reduce the computing time of the similarity-scoring module in terms of satisfying the time constraint of a real-time system.

In this paper, we develop both a multiple core computing algorithm and a distrubited algorithm to speedup the performance of the RT-PSM program. The comparison is made betweet our algorithm and the RT-PSM algorithm in [1]. The definition of sensitivity and specificity in [1] refer from the textbook [11], which are different from currently widely accepted formula. We ignore the name of sensitivity and spedificity, but employ the evalutation formula used in [1] to carry out our comparisons.

### The similarity-scoring module of the RT-PSM program

Given an experimental spectrum, the similarity-scoring module searches the database of candidate peptides to find the best matched one according to a similarity score. The similarity score is calculated by comparing the difference of m/z values of ions between the experimental MS/MS spectrum and a theoretical spectrum of a peptide in the candidate database. Eight kinds of fragment ions are considered in the RT-PSM program, which are listed in Table 3.

**Table 3:** **Types of fragment ions and their m**/**z values in the RT**-**PSM program**

**Ion type**	**m/z value**
b^+^	b
b^+^-H_2_O	b-18
b^+^-NH_3_	b-17
b^+^-CO(a^+^)	b-28
y^+^	y
y^+^-H_2_O	y-18
y^+^-NH_3_	y-17
y^+^-NH(z^+^)	y-15

The signs ‘+’ in superscript of the letter b (and y) denote the singly charge positive ions. The symbol b (and y) without any superscript denote the m/z value of a b-(or y-)ion with a single positive charge. The symbol ‘-H_2_O’, ‘-NH_3_’, ‘-CO’ and ‘-NH’ represent an ion lose a small molecule of ‘H_2_O’, ‘NH_3_’, ‘CO’ and ‘NH’, respectively.

Generally, it is not necessary to search the whole database for finding the best-matched candidate peptide. The mass difference between an experimental peptide and its matched candidate peptide is often very small. A nearest neighbor search (NNS) is employed in the RT-PSM algorithm. Suppose M_m_ is the mass of an experimental peptide and t is the tolerance range of the NNS. Only those candidate peptides with mass range between M_m_ - t and M_m_ + t need to be considered. The RT-PSM program proposed by Wu et al. [1] employs the most common binary search method to perform the NNS [12]. The time complexity of the binary search is O (log n). Hence, the time spending on the peptide search is related to the size of peptide database.

**Figure 5:**
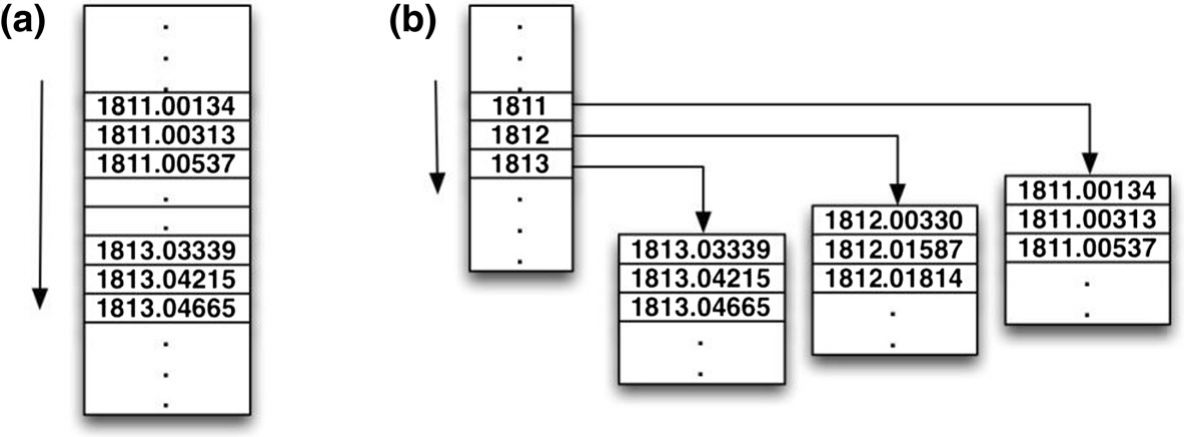
**An improved data structure for searching peptide database. (a)** The original Ppptide data structure in the form of a large array; **(b)** The improved peptide data structure with the integer part of the mass value as indices.

In this study, we propose to employ an 2-Dimensional peptide database search method to decrease the searching time. The method is described as follows. First, instead of treading the whole peptide database as a large array, the database is separated into a series of small peptide groups (indices) according to the integer part of the peptide mass. After that, each subset is indexed according to the integer part of the mass value. The integer peptide database is a sorted collection containing indexed sub-databases as shown in Figure 5.

With this improved data structure of peptide database, the peptide searching consists of two steps. The first step is to search if the integer part X of the target peptide mass with tolerance value t is indexed by the peptide database (X ± t). If the value is found, then the first record in the indexed sub-array is the matched peptide, and the time complexity of this step is O(2 t). If the first step cannot find a matched peptide and the database also contains a subset with index (X-1), then the second step is using the binary search method to check if this subset contains the matched peptide. The time complexity of the second step is O(log(subset length)). The pseudo code of the 2-dimensional peptide database search method for peptide database searching is shown in Algorithm 1.

In terms of the time consuming for each peptide P in the candidate peptide group, the scoring time t_k_ is.

$$t_{k}=\sum_{i=1}^{n}\left(t_{1i}+t_{2i}\right),$$

where n is the number of ion types that are considered in the algorithm, t_li_ is the peptide searching time, t_2i_ is the peptide scoring time. For each candidate peptide group, the total time of the similarity-scoring module is

$$t_{\mathrm{total}}=\sum_{k=1}^{N}t_{k},$$

where N is the number of peptides in the group.

Algorithm 1: 2-Dimensional Peptide Database Search Method

### Multi-core Computing and Distributed Computing

The similarity-scoring module in the RT-PSM program is a typical CPU-bound computation function, which means the computing time of the function is determined principally by the speed of CPU. Normally, one processor can only execute one function at one time. In order to reduce the time consumed for the similarity-scoring module, we propose a parallel algorithm that combines the advantages of multi-core computing and distributed computing to achieve the maximum performance.

### The multi-core RT-PSM (MC RT-PSM) algorithm

**Figure 6:**
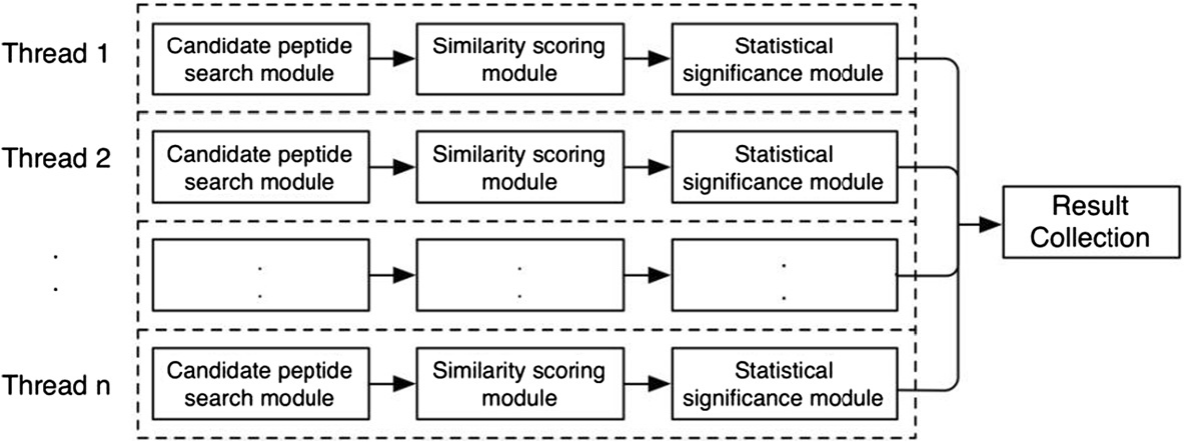
**The workflow of the MC RT-PSM program.**

The MC RT-PSM is based on the Hyper-Threading Technology (HT Technology), which is a form of simultaneous multi-threading that takes advantage of super scalar architecture (multiple instructions operating on separate data in parallel) [13]. Based on this technology, the CPU-bound computations can execute multiple scoring functions concurrently in a single-CPU workstation [14]. The workflow of the MC RT-PSM program is illustrated in Figure 6.

The maximum number of threads can be used in the MC RT-PSM is based on the number of logical processors. The pseudo code of MC RT-PSM algorithm is shown in Algorithm 2.

Algorithm 2: The pseudo code of MC RT-PSM algorithm

### The distributed computing RT-PSM (DC RT-PSM) algorithm

Similar to MC RT-PSM algorithm, the DC RT-PSM algorithm also needs to separate a large task into several sub-tasks and executes them concurrently. However, they are different in the following two aspects. Firstly, the DC RT-PSM algorithm is designed to run on a distributed computer, such as a computer cluster, rather than a single-CPU workstation. The cluster is a computer system with the processing elements connected as a network. The Windows HPC SDK package provides a stable and user-friendly development environment for us to develop the program of the DC RT-PSM algorithm. Secondly, each processor has its own memory in the DC RT-PSM program, while all processors access to a shared memory in the MC RT-PSM program [15].

In our case of the DC RT-PSM algorithm, the whole identification procedure is divided into several sub RT-PSM tasks. Results of those computations are combined by a head node [16]. Each sub task runs in an individual worker node of the cluster. In order to achieve the minimum execution time, the head node creates, distributes, synchronizes and monitors tasks in each worker node. The pseudo code of the distributed task management algorithm for the head node is shown in Algorithm 3.

Algorithm 3: The pseudo code of DC RT-PSM algorithm

**Figure 7:**
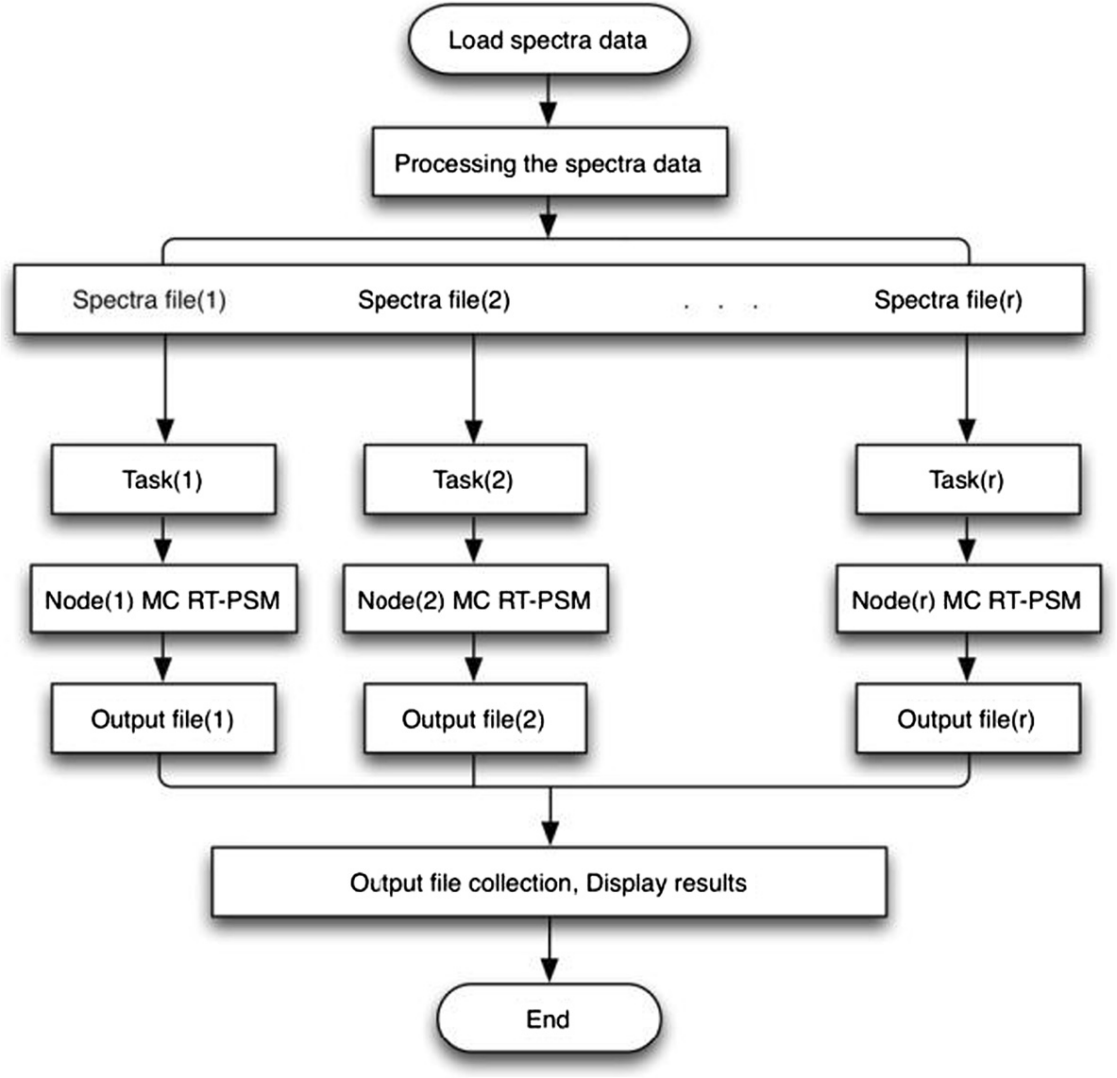
The workflow of the distributed task management program.

The DC RT-PSM algorithm consists of three steps. Firstly, it loads the experimental MS/MS spectral data file and divides it into a specified number of small files. Each smaller file is assigned to a related worker node. Secondly, it creates a sub RT-PSM task for each worker node and starts all tasks simultaneously. The DC RT-PSM monitors all tasks when they are executing. It collects the feedback information, including the task status and exceptions. Finally, after all tasks are accomplished, the DC RT-PSM collects results from each worker node and generates a final report as the output. Figure 7 shows the workflow of the distributed task management program.

The time consumption of the similarity scoring function in DC RT-PSM algorithm is:

$$t_{j}=t_{1j}+t_{2j}+t_{3j},j=1,2,\ldots ,r$$

where j is the number of worker node, t_1j_, t_2j_ and t_3j_ are the peptide searching time, the peptide scoring time and the message communication time spend in the J^th^ worker node. In practice, the time consuming of message communication in each thread is fixed. Hence, when processing a large dataset, the peptide searching time t_1j_ and the peptide scoring time t_2j_ contribute a large amount of time consuming compared with the message communication time. The total time consumption of the DC RT-PSM algorithm is:

$$t_{\text{total}}^{'}=t_{o}+\max\left\{t_{j}\right\},j=1,2,\ldots ,r.$$

where t_o_ is the task initial time.

## Abbreviations

PSMs: Peptide-spectrum matches; RT-PSM: Real-time peptide-spectrum matching; MS/MS: Tandem mass spectra; MC RT-PSM: Multi-core RT-PSM; DC RT-PSM: Distributed computing RT-PSM; PVM: Parallel Virtual Machine; MPI: Message Passing Interface; NNS: Nearest neighbor search; HT Technology: Hyper-Threading Technology.

## Competing interests

The authors declared that they have no competing interest.

## Authors’ contributions

FXW initiated this study. BC, JS and FXW discussed the algorithms and their implemetnations. JS performed the experiments, BC and JS drafted the manscript. FXW and BC revised the manscript substaintially. All authors read and approved the final manuscript.

## Supplementary Material

Additional file 1 Source code of the DC RT-PSM algorithm and sample datasets. Click here for file
